# Highly Efficient Catalysis of Sulfur Reduction Reaction: 3*d* ^10^‐Based Catalysts

**DOI:** 10.1002/advs.202508473

**Published:** 2025-07-17

**Authors:** Kangxin Chen, Haili Luo, Yuanyuan Chang, Daying Guo, Yuchuan Zhu, Longyang Zhou, Xi'an Chen, Shun Wang

**Affiliations:** ^1^ College of Chemistry and Materials Engineering Wenzhou University Wenzhou 325035 China; ^2^ Institute of Materials Science and Devices School of Materials Science and Engineering Suzhou University of Science and Technology Suzhou 215009 China

**Keywords:** 3d^10^‐based materials, catalysts, lithium‐sulfur batteries, polysulfide shuttling

## Abstract

Lithium‐sulfur batteries (LSBs), with their high energy density compared to lithium‐ion batteries, are now strong candidates for next‐generation energy storage systems. However, insufficient redox kinetics leads to shuttle effect and lithium dendrites, which hinder their commercial application. In this paper, the latest progress of 3d^10^‐based materials such as copper, zinc and their composites as cathode materials for LSBs is discussed, and the kinetics of polysulfide reduction catalyzed by copper‐zinc‐based materials and the mechanism of restraining shuttle effect are discussed in detail. Several strategies for the rational design of 3d^10^‐based catalysts to improve the polysulfide reduction reaction are summarized. Finally, we summarize the challenges faced by 3d^10^‐based materials in LSBs applications and present an outlook to improve the reference for the design of next‐generation LSBs materials.

## Graphical abstract

Taking Cu and Zn, two typical 3d^10^ metal elements, as an entry point, this paper reviews the application of Cu‐based materials, Zn‐based materials and their Cu‐Zn‐based materials in accelerating the sulfur reduction reaction of lithium‐sulfur batteries, which can effectively alleviate the three major problems faced by lithium‐sulfur battery cathodes, and show significant advantages in improving the overall performance of the batteries.

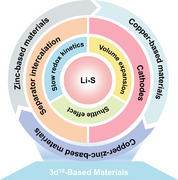

## 1 Introduction

Lithium‐ion batteries are the most successful and widely used energy storage system with energy densities increasing to about 300 Wh kg^−1^. However, their development is limited by the theoretical capacity of layered lithium transition metal oxide cathode, and we urgently need to find a new generation of battery systems with high energy density.^[^ ^1^, ^2^, ^3^, ^4^, ^5^, ^6^ ^]^ Lithium‐sulfur batteries (LSBs) are based on the multi‐electron redox reaction of sulfur (S). They break through the limitations of lithium‐ion batteries with inserted cathode and graphite anodes to achieve higher energy density, and is one of the most promising next‐generation battery systems.^[^ ^7^, ^8^ ^]^ Although LSBs exhibit a high theoretical specific capacity of 1675 mAh g^−1^, there are still many problems on the cathode side of the battery, seriously hindering its commercial application.^[^ ^9^, ^10^, ^11^, ^12^ ^]^ Due to the poor conductivity of S and its discharge product lithium sulfide (Li_2_S), it can significantly slow down charge transfer and redox reaction processes on the cathode side.^[^ ^13^, ^14^, ^15^ ^]^ In addition, during the charging and discharging process, lithium polysulfides (LiPSs) migrate from the cathode to the anode and back to the cathode driven by the concentration gradient and the internal electric field, forming a “shuttle effect.” This shuttle effect leads to irreversible loss of active material and exacerbates capacity decay.^[^ ^16^, ^17^, ^18^ ^]^ Since the density of S is 2.07 g cm^−3^ while the density of Li_2_S is 1.66 g cm^−3^, the difference in their densities leads to a volume expansion of about 80% during charging and discharging, resulting in electrode pulverization.^[^ ^19^, ^20^ ^]^ All these problems are due to the slow kinetics of sulfur reduction reaction (SRR). To address these issues, it has been found that carbon materials such as carbon nano‐tubes (CNTs)^[^ ^21^, ^22^ ^]^ carbon nanofibers (CNF),^[^ ^23^ ^]^ graphene,^[^ ^24^ ^]^ and porous carbon (PC)^[^ ^25^, ^26^ ^]^ used as S hosts can significantly improve the electrical conductivity of the cathode and inhibit LiPSs shuttling by physically limiting the domain. However, nonpolar carbon materials have insufficient affinity for LiPSs, by contrast, transition metal materials tend to have higher polarity and excellent catalytic activity. Currently, transition metal oxides,^[^ ^27^ ^]^ sulfides,^[^ ^28^ ^]^ nitrides,^[^ ^29^ ^]^ phosphides,^[^ ^30^, ^31^ ^]^ and carbides^[^ ^32^, ^33^ ^]^ have been widely used in LSBs, and unlike the physical adsorption of carbon materials, they usually inhibit the shuttling of LiPSs through strong polar interactions.

Among transition metal compounds, 3d^10^‐based materials exhibit excellent catalytic properties due to their unique d‐orbital electronic configurations. Specifically, the d‐orbital of Copper (Cu) can be d‐p hybridized with the p‐orbital of S to optimize the charge transfer path, reduce the interfacial impedance, and accelerate the kinetics of the S reduction reaction (SRR). It can be compounded with S to form a three dimensional (3D) conductive framework, effectively enhancing the charge transfer efficiency of the S cathode and alleviating the insulating properties of S. Moreover, some Cu‐based materials can inhibit LiPSs shuttling and improve the cycling performance of LSBs through chemical anchoring of surface oxygen functional groups or physically limiting domains of porous structures.^[^ ^34^, ^35^ ^]^ Zinc‐based materials have similar advantages to copper‐based materials in that the 3d orbitals of Zinc (Zn) can also form d‐p hybridization with the 3p orbitals of S to construct continuous charge transfer channels. Meanwhile, it has a higher d‐band center position, reducing the activation energy of the reaction and making it more catalytically active. Moreover, the 3d^10^ electronic configuration of Zn generates a localized polarization field that enhances the anchoring ability to LiPSs, and this electronic configuration contributes to the improvement of LSBs performance.^[^ ^36^, ^37^ ^]^ The synergistic effect of Cu and Zn can optimize the electron transfer path, and the strong polarity of the Zn‐S bond can induce the directional migration of electrons from Zn to Cu, significantly improving the SRR kinetics of LSBs. In addition, the two can form catalytic active centers through d‐p orbital coupling to reduce the activation energy of S‐S bond rupture, and the reversible change of oxidation state during charging and discharging can effectively improve the cycling performance of the battery.^[^ ^38^, ^39^, ^40^ ^]^ Although other 3d^10^‐based homologous metals have similar structures to Cu and Zn, they have significant drawbacks that severely limit their application in LSBs. Toxic cadmium poses environmental risks, liquid mercury prevents the formation of stable electrode interfaces, costly silver/gold metals hinder large‐scale use, and all exhibit consistently lower catalytic activity than Cu and Zn. In contrast, Cu and Zn are more advantageous in terms of cost, environmental friendliness, and catalytic activity. In this review, we review the relationship between the crystal structure and electrochemical performance of 3d^10^‐based materials, such as Cu‐based, Zn‐based and Cu‐Zn‐based materials as well as the ways to enhance the electrochemical performance of the materials. Finally, the future development trend of 3d^10^‐based materials in high‐performance lithium‐sulfur battery (LSB) is prospected. This review will provide beneficial enlightenment for the design and preparation of high‐performance LSBs.

## 2 Design Principles for 3d^10^‐Based Catalysts

**Figure 1:**
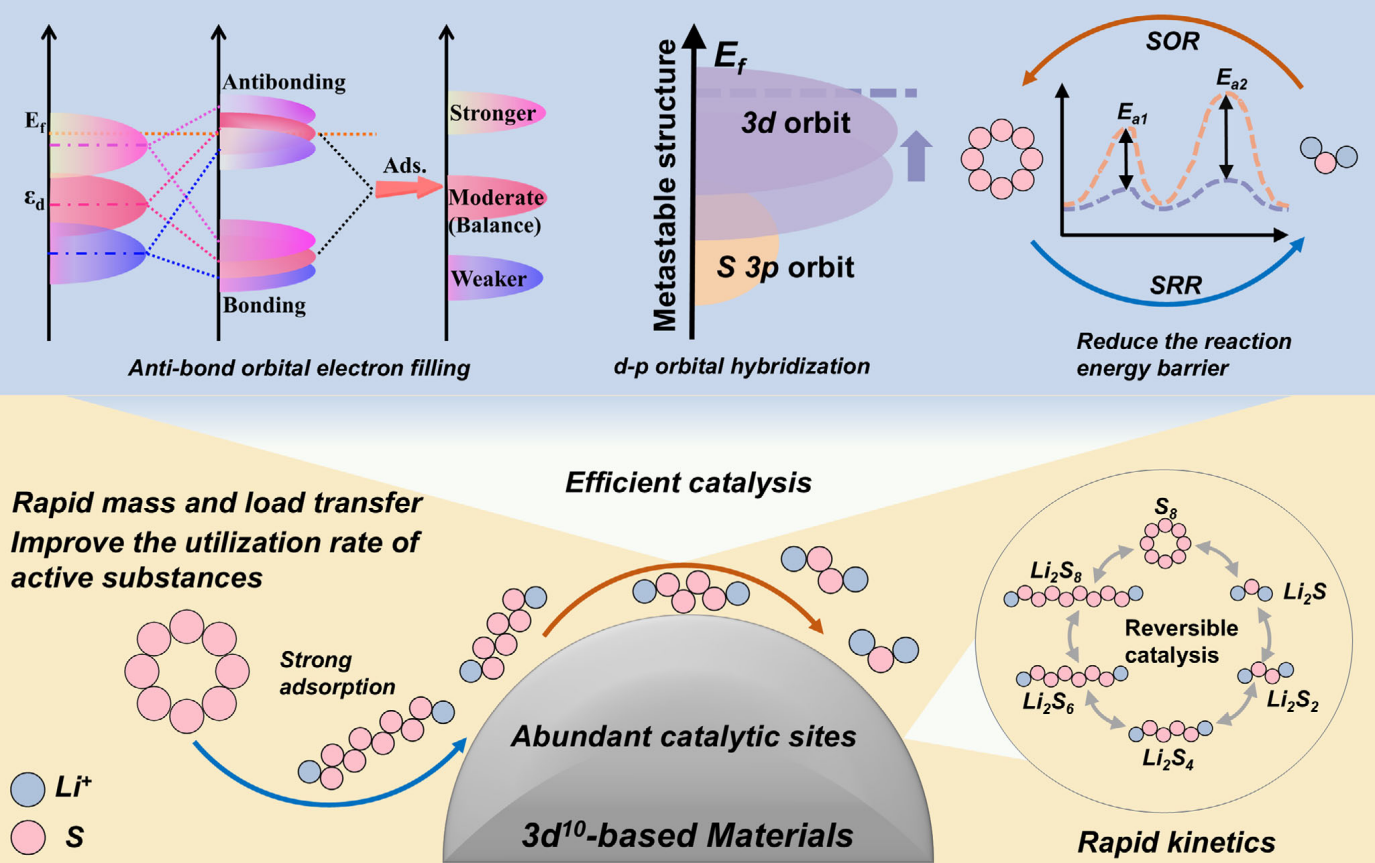
Schematic representation of the accelerated SRR reaction of Cu‐Zn based materials.

To address the inherent limitations of the shuttle effect and slow redox kinetics of LiPSs in LSBs, the researchers designed a 3d^10^‐based metal catalyst. The design aims to enhance the overall battery performance by providing moderate LiPSs adsorption capacity, accelerating LiPSs conversion, and promoting rapid Li_2_S desorption (**Figure** **1**).

The adsorption of LiPSs by the catalyst is crucial to mitigate the shuttle effect, but too strong adsorption may not only hinder the reaction kinetics, but also lead to excessive binding of the discharge product Li_2_S. Therefore, the optimal catalytic performance needs to follow the “Sabatier” principle in multiphase catalysis, that is, the interaction between the catalyst and the reactants should be just right, neither too strong nor too weak.^[^ ^41^ ^]^ In 3d^10^‐based metal catalysts, the covalent bonds formed between their d orbitals and the p orbitals of sulfur help to suppress the shuttle effect. Specifically, the catalyst d orbitals and the S 3p orbitals will spatially form σ‐bonds, while the lower unoccupied d‐band energy level facilitates the acceptance of electrons from LiPSs for enhanced adsorption. The key to the design is to achieve a moderate p‐d orbital overlap, thus striking a balance between adsorption strength and reaction kinetics.^[^ ^42^, ^43^ ^]^

Based on density of states (DOS) analysis, the core strategy of 3d^10^ metal catalyst design is to modulate the localized electronic states around the Fermi energy level (E_f_) in order to simultaneously optimize the adsorption and conversion kinetics.^[^ ^44^ ^]^ Since the d‐band of the 3d^10^‐based genus itself is far from E_f_, it is usually necessary to introduce a high local density of states in the vicinity of E_f_ by defect engineering, doping, or constructing heterostructures. Since the d‐band of the 3d^10^‐based genus itself is far from E_f_, it is usually necessary to introduce a high local density of states in the vicinity of E_f_ by defect engineering, doping, or constructing heterostructures. These introduced densities of states act as active sites to enhance the anchoring effect on LiPSs by promoting charge transfer. In addition, rational energy band modulation can also lower the electron transfer energy barrier and accelerate Li_2_S deposition/dissolution, thus accelerating the Li_2_S oxidation reaction (SOR). In summary, the design of 3d^10^‐based catalysts centers on the precise construction of high local density of states near E_f_ using defect, doping, or interfacial engineering.^[^ ^45^, ^46^, ^47^ ^]^ This strategy can endow the catalyst with moderate LiPSs adsorption capacity and efficient electron transfer efficiency while maintaining the stability of the material system stability system, ultimately realizing the effective catalytic LiPSs conversion and suppressing the shuttle effect.

## 3 Copper‐Based Catalysts Materials for LSBs

Cu‐based materials are rich in lone‐pair electrons and have unfilled d‐orbitals, facilitating the enhancement of the catalytic activity of the materials themselves through d‐band center shifts as well as the tuning of the electron spin states.^[^ ^48^ ^]^ Meanwhile, the copper‐based materials have lithophilic/sulfofilic sites that can effectively adsorb LiPSs. The position of the d‐band center of the catalyst will affect its adsorption strength for the reactants.^[^ ^49^, ^50^ ^]^ Cu has a full d‐orbital, and its d‐band center position is positioned relatively low. The low d‐band center means that the adsorption strength of LiPSs at the Cu site is usually weaker than that on transition metals with partially filled d‐orbital, whose d‐band centers of these metals are usually high and which adsorb LiPSs very strongly. According to the “Sabatier” principle, the catalytic conversion of LiPSs requires moderate adsorption strengths; adsorption that is either too weak or too strong is detrimental to this conversion.^[^ ^51^, ^52^ ^]^ Cu provides a moderate adsorption strength, which can effectively adsorb and activate the “S‐S” bonds in LiPSs without blocking the active sites due to strong adsorption. In this section, several types of typical Cu‐based materials will be discussed to analyze the relationship between their structures and functional mechanisms.

### 3.1 Copper Oxide Catalysts

**Figure 2:**
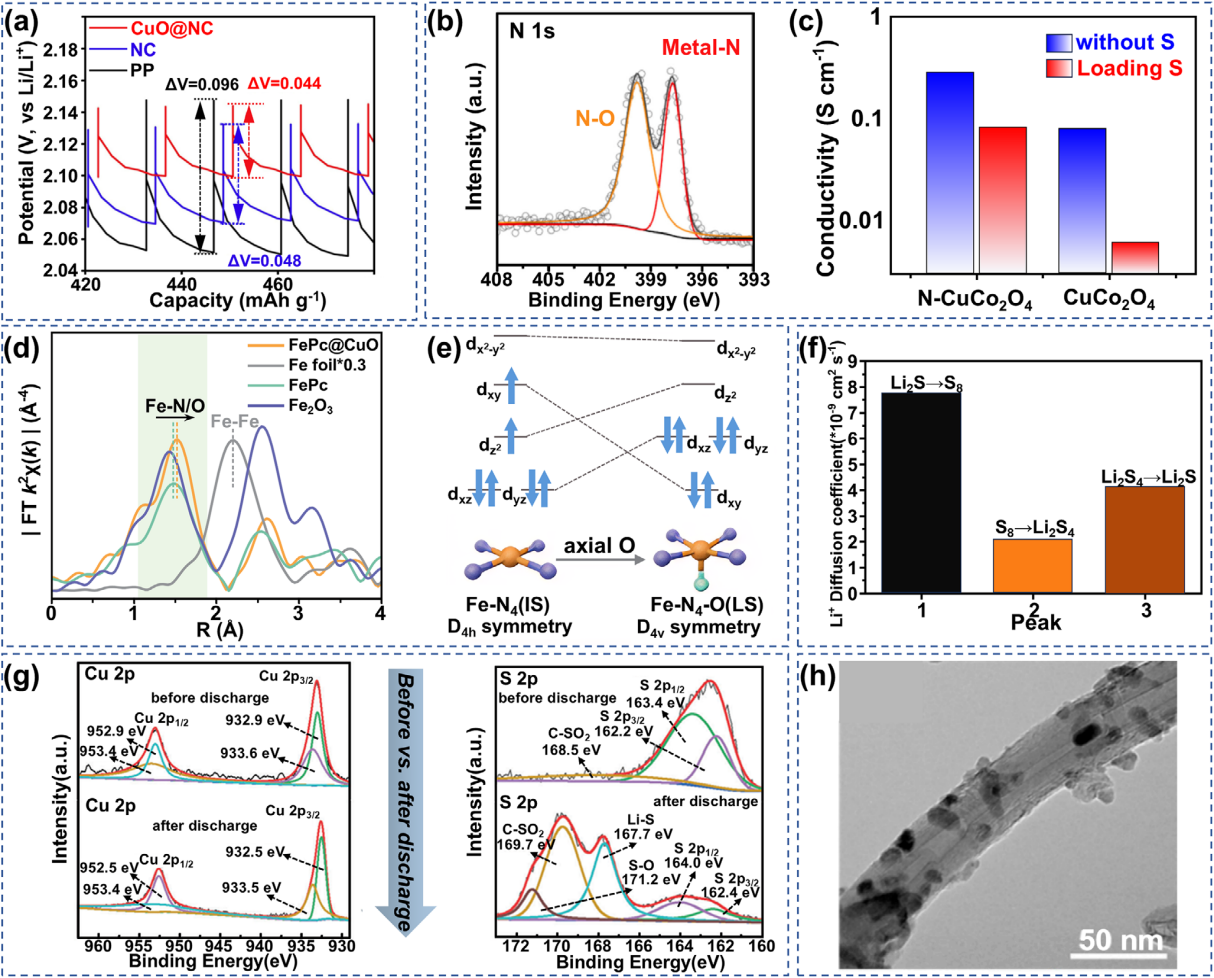
a) Localized enlargement of GITT curves for PP, NC and CuO@NC separator cells. Reproduced with permission.^[^ ^53^ ^]^ Copyright 2025, Elsevier. b) High‐resolution XPS spectrum of N 1s. c) Electrical conductivity of N‐CuCo_2_O_4_, CuCo_2_O_4_ and their S composites. (b,c) Reproduced with permission.^[^ ^54^ ^]^ Copyright 2021, Elsevier. d) R‐space FT‐EXAFS spectra of Fe foil, Fe_2_O_3_ and FePc@CuO. e) Fe 3d orbital arrangement. (d,e) Reproduced with permission.^[^ ^55^ ^]^ Copyright 2024, Wiley‐VCH. f) Li^+^ diffusion coefficient of μ‐cubes‐S. Reproduced with permission.^[^ ^56^ ^]^ Copyright 2021, Elsevier. g) XPS plots of Cu 2p and S 2p before and after charge and discharge. Reproduced with permission.^[^ ^57^ ^]^ Copyright 2022, Wiley‐VCH. h) TEM image of CuCo_2_S_4_‐1/CNT. Reproduced with permission.^[^ ^58^ ^]^ Copyright 2022, MDPI.

Cu oxide (CuO) as a typical metal oxide can anchor LiPSs via polarity‐polarity interactions. It has been shown that CuO maintains both excellent adsorption properties and significantly enhances the SRR kinetics under the synergistic effect of the nitrogen‐doped carbon (NC) skeleton. For example, Li's team developed CuO@NC modified separator materials and found that the relaxation voltage of the CuO@NC system was significantly lower compared to that of traditional modified separators under the same discharge depth by constant current gap titration technique (GITT) system analyses, indicating the superior ion transport properties of CuO@NC and demonstrating its lower polarization and enhanced SRR kinetics (**Figure** **2**a).^[^ ^53^ ^]^

Besides, S host materials enable improved LiPSs conversion kinetics driven by nitrogen‐doped synergistic interfacial optimization strategies. For example, Pu research team has innovatively developed nitrogen‐doped double‐shell‐layered CuCo_2_O_4_ hollow nanospheres (N‐CuCo_2_O_4_), X‐ray Photoelectron spectroscopy (XPS) confirmed that N exists in the form of metal‐N bonds in oxygen vacancies and its lattice structure, and this doping contributes to lattice stability (Figure 2b).^[^ ^54^ ^]^ Four‐probe tests showed that N doping significantly improved the electrical conductivity of the composites, and the composite of N‐CuCo_2_O_4_ with S still maintained excellent conductive properties. This structural advantage could promote the SRR kinetic process of LiPSs (Figure 2c). Although CuO lacks catalytic properties on its own, it can be used as a substrate material to anchor other molecules. Moreover, Chen and colleagues precisely anchored iron titanocene (FePc) to the CuO surface by axial covalent bonding. The terminal oxygen on the CuO surface formed a covalent bond with the Fe center of the FePc molecule, and this oxygen coordination induced a shift in the spin state of Fe from medium to low spin. This d‐orbital optimization enhanced adsorption energy (Figure 2d,e).^[^ ^55^ ^]^ These structural changes allowed Li‐S cells with FePc@CuO groups to show excellent cycling stability at 2C. Inspired by this method, they enclosed a series of but dispersed transition metal titanocenes on CuO, and similar electronic configuration evolution patterns were observed in metal catalyst systems such as Mn, Co, and Ni.

### 3.2 Copper Sulfide Catalysts

The unoccupied d‐orbitals of Cu and the lone pair of electrons of S can form a strong chemical anchoring with lithium (Li) and S in LiPSs, and Cu sulfide (CuS) itself has a low Li^+^ diffusion energy barrier, favoring Li^+^ diffusion.^[^ ^59^ ^]^ This capability is based on the strong chemisorption and excellent SRR catalytic activity of CuS. For example, Long's team successfully developed CuS@void@Co_3_O_4_ bimicrocubic structures (μ‐cubes) with dual limiting domains. This cathode material exhibited three typical redox peaks at a scan rate of 0.1 mV s^−1^, and the corresponding Li^+^ diffusion coefficients indicated that μ‐cubes‐S promotes the conversion of Li_2_S_4_ to Li_2_S (Figure 2f).^[^ ^56^ ^]^ Similarly, Geng's team revealed a strong immobilization mechanism of polysulfides by CuS through hierarchical structure design. They constructed CuS/CNTs materials with hierarchical structures by compositing coral‐like CuS with highly conductive carbon nanotubes.^[^ ^57^ ^]^ XPS indicated that before and after discharge, the binding energy of Cu 2p orbitals shifted negatively, while that of S 2p orbitals shifted positively. This might be due to the charge transferred between LiPSs anions and Cu^2+^. Notably, the newly emerged characteristic peak at 171.2 eV confirmed the formation of S─O bonds, further corroborating the strong immobilization mechanism of LiPSs by CuS (Figure 2g). Based on the fast electron transport channel provided by carbon materials, CuS enhances the anchoring of S species through chemical bonding. This approach enabled the battery to exhibit considerable capacity and stable cycling performance and provided ideas for the modification of LSBs. Compared with monometallic sulfide systems, bimetallic sulfides further optimize the synergistic mechanism of adsorption and catalysis through component modulation. Wang's research team constructed CuCo_2_S_4_‐modified carbon nanotube (CNT) composite systems through a hydrothermal synthesis strategy.^[^ ^58^ ^]^ By adjusting the ratio of precursors, CuCo_2_S_4_ was successfully anchored to CNTs. This structural feature synergistically enhanced the chemisorption efficiency of the composites on LiPSs and fully exposed the SRR catalytically active sites (Figure 2h). However, some oxygen‐containing groups are generated on the surface of CNT, and these groups might affect the loading of CuCo_2_S_4_ and the charge transport kinetics of the battery system. Despite its good conductivity, excellent adsorption properties and high specific capacity presented in the cell, CuS is not sufficiently catalytic for Li_2_S_6_→Li_2_S_2_/Li_2_S conversion compared to CoS_2_.^[^ ^60^ ^]^ In the future, optimization may still be required through methods such as composite structures or defect engineering.

### 3.3 Copper Selenide Catalysts

**Figure 3:**
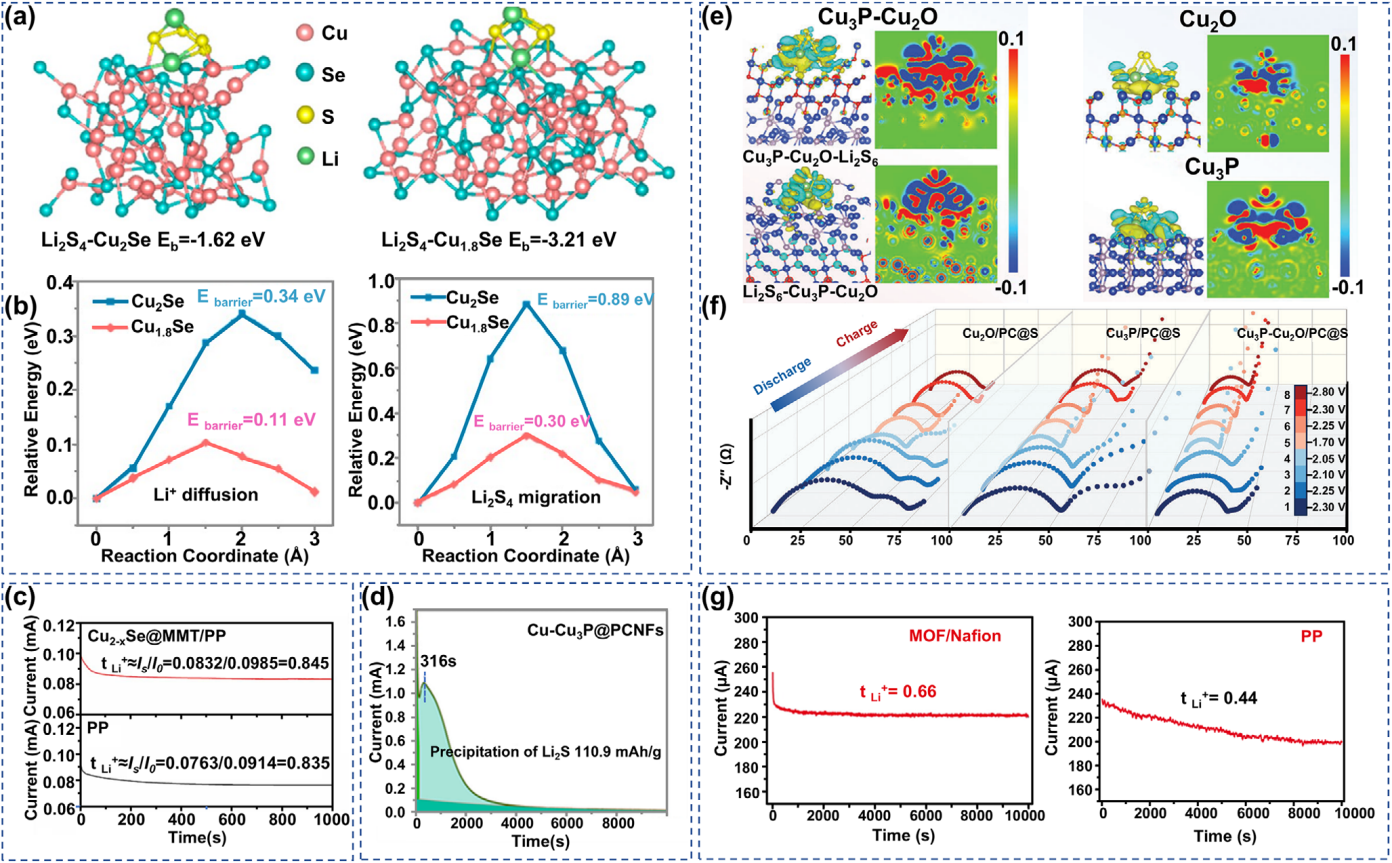
a) Adsorption configurations of LiPSs on Cu_2_Se (220) and Cu_1.8_Se (111) crystal faces. b) Diffusion energy barriers of Li^+^ with Li_2_S_4_ at Cu_2_Se and Cu_1.8_Se crystal faces. a,b) Reproduced with permission.^[^ ^68^ ^]^ Copyright 2022, American Chemical Society. c) Li^+^ diffusion properties of Cu_2‐x_Se@MMT/PP and PP separators. Reproduced with permission.^[^ ^70^ ^]^ Copyright 2024, Elsevier. d) Li_2_S nucleation kinetics of Cu‐Cu_3_P@PCNFs. Reproduced with permission.^[^ ^71^ ^]^ Copyright 2024, Elsevier. e) Charge density variation of Cu_2_O/PC@S, Cu_3_P/PC@S and Cu_3_P─Cu_2_O/PC@S on Li_2_S_6_. f) In situ EIS maps of Cu_2_O/PC@S, Cu_3_P/PC@S and Cu_3_P─Cu_2_O/PC@S. (e,f) Reproduced with permission.^[^ ^72^ ^]^ Copyright 2025, Wiley‐VCH. g) Li^+^ diffusion in MOF/Nafion and PP separators. Reproduced with permission.^[^ ^73^ ^]^ Copyright 2022, Elsevier.

Similar to their cousins, oxides and sulfides, transition metal selenides (TMSe) not only inherit the chemical affinity of polar interfaces, but their unique layered structure and tunable electron cloud distribution properties also endow them with excellent charge transfer kinetics and chemical stability.^[^ ^61^, ^62^, ^63^ ^]^ Through the synergistic coordination between selenium atoms and metal atoms, these materials form abundant surface active sites and continuous electron transport channels, effectively promoting the charge transfer and ion diffusion processes in SRR.^[^ ^64^, ^65^, ^66^ ^]^ It is particularly noteworthy that in the Cu_2‐x_Se material system, Cu vacancies, as a key defect type, play a significant role in modulating the electronic structure and catalytic properties of the material. These vacancies essentially act as p‐type dopants, introducing positively charged centers in the lattice, thereby significantly enhancing the hole carriers and conductivity of the material and optimizing the charge transport efficiency at the surface. More importantly, the presence of Cu vacancies can induce local electronic states and regulate the Fermi level position, directly affecting the adsorption strength of the material surface for reaction intermediates, thereby optimizing its catalytic reaction path and activity.^[^ ^67^ ^]^ Based on an in‐depth understanding of the mechanism of Cu vacancy action, Yang's team successfully synthesized cubic‐phase Cu_1.8_Se catalysts with copper vacancy defects using a phase engineering strategy.^[^ ^68^ ^]^ Theoretical simulations showed that in the adsorption configuration of Li_2_S_4_, S preferentially formed S‐Cu bonds with Cu sites, while Li generated strong Li‐Se interactions with Se sites. Notably, Cu_1.8_Se exhibited better binding energy for Li_2_S_4_ compared to Cu_2_Se, a result that was consistent with adsorption tests (**Figure** **3**a). Further studies revealed that the introduction of Cu vacancies significantly optimized the electronic structure of the material, lowered the migration energy barriers of Li^+^ and Li_2_S_4_, and promoted Li^+^ diffusion and Li_2_S_4_ surface migration (Figure 3b). The introduction of this metal vacancy not only enhanced the chemical anchoring ability of LiPSs, but also establishes an efficient ion transport channel. Similarly, Yuan's group designed a material composed of graphene oxide and Cu_2‐x_Se, offering abundant sulfofilic active sites and providing ideas for building 2D TMSe materials for LSBs.^[^ ^69^ ^]^ Based on the above 2D material design ideas, Wu et al. synthesized Cu_2‐x_Se@montmorillonite (Cu_2‐x_Se@MMT) heterostructures with a layered structure using a one‐pot wet chemical method.^[^ ^70^ ^]^ The Li^+^ migration number of Cu_2‐x_Se@MMT modified separator was found to be higher than that of pristine polypropylene(PP) separator using chrono‐current method (Figure 3c). This performance improvement stemmed from the anchoring effect of the MMT layered matrix in the heterostructure on the anions in the electrolyte and the optimization of the Li^+^ transport path by Cu_2‐x_Se. The study of copper selenide materials in LSBs is still at an early stage, but their well‐balanced properties show unique advantages. Compared with the lower‐conductivity sulfides and transition metal tellurides, Cu selenide overcomes the charge transport dilemma of sulfide cathodes while reducing the risk of mechanical failure, providing a new direction for improving battery energy density over cycle life.

### 3.4 Other Copper‐Based Catalysts

In addition to the above materials, copper phosphide (Cu_3_P), Cu─MOFs and Cu single‐atom catalysts (SACs), with their unique electron transport properties and SRR multistep reaction modulation capabilities, have shown significant potential for application in LSBs systems. The current study focuses on the construction of a multidimensional adsorption‐conversion synergistic mechanism for Cu_3_P and a coordination environmental engineering strategy for Cu single atoms (SA). These atomic‐level structural optimizations provide ideas for advancing the kinetics of the SRR with cyclic stability.

#### 3.4.1 Copper Phosphate Catalysts

Transition metal phosphides are characterized by a contracted energy band distribution due to the moderate electronegativity and coordination flexibility of the (P) phosphorus atoms in their structure, contributing to a significant enhancement of the metal‐P bond orbital coupling degree. This electronic reconfiguration effect not only optimizes the charge migration efficiency, but also lowers the SRR conversion energy barrier of LiPSs through surface electron density modulation, thus enhancing the redox efficiency and active substance utilization of S.^[^ ^74^, ^75^, ^76^ ^]^ Based on this electronic structure modulation strategy, the researchers applied the synergistic design of porous skeleton and polar components to the cathode construction. For example, Guo et al. have innovatively synthesized pollen‐derived porous carbon/copper phosphide (PC/Cu_3_P) hybrids. P‐ and N‐*co*‐doped porous PCs could be obtained after pollen carbonization.^[^ ^77^ ^]^ Biochar/Cu_3_P material hybrids promoted S loading through porous structure and regulated the adsorption process of LiPSs efficiently and rapidly with the help of Cu_3_P component, providing inspiration in the field of electrochemical energy storage. Constructing heterogeneous structures is another effective method to enhance the performance of materials. The built‐in electric fields (BIEF) formed between interfaces can improve the charge transfer at the interface and strengthen the SRR kinetics.^[^ ^78^ ^]^ For example, Zhu's group developed Cu─Cu_3_P heterostructure nanoparticles embedded in hierarchical porous carbon nanofibers. The material significantly enhanced the chemical anchoring of LiPSs by Cu_3_P and accelerated the nucleation rate of Li_2_S through the spontaneous BIEF formed at the heterogeneous interface. This heterogeneous structure effectively improved the cycling stability of the battery (Figure 3d).^[^ ^71^ ^]^ It was shown that the Cu_3_P/metal oxide heterogeneous interface could significantly enhance the conversion kinetics of LiPSs through two‐site synergy. Recently, Zhu's team prepared non‐homogeneous Cu_3_P─Cu_2_O nanoparticles loaded in porous carbon (Cu_3_P─Cu_2_O/PC) using a one‐step thermal treatment method.^[^ ^72^ ^]^ The charge density difference between Cu_3_P and Cu_2_O triggered a strong electron enrichment at the interface of the heterostructure, and this interfacial coupling effect helped to accelerate the charge transfer between Cu_3_P─Cu_2_O and LiPSs. Further studies showed that the interfacial coupling effect prompted the generation of Cu‐S bonds between Cu and polysulfide anions to realize the efficient anchoring of LiPSs (Figure 3e). Meanwhile, in situ EIS analysis revealed that the Cu_3_P─Cu_2_O/PC@S cathode exhibited a significantly lower charge transfer resistance (R_ct_) compared to the Cu_3_P/PC@S and Cu_2_O/PC@S systems; notably, the R_ct_ of this cathode did not change significantly during charging, a phenomenon that verified the excellent stability of its solid‐liquid‐solid phase transition process (Figure 3f). The study of Cu_3_P materials in LSBs is still at an early stage, and their unique charge transport properties and structural stability give them a significant advantage as S hosts. The multidimensional active interface network optimized by nano‐configuration can synchronously enhance the S reaction kinetics and the domain‐limiting ability of LiPSs, further breaking through the performance bottleneck of LSBs.

#### 3.4.2 Copper Metal‐Organic Framework Materials Catalysts

**Figure 4:**
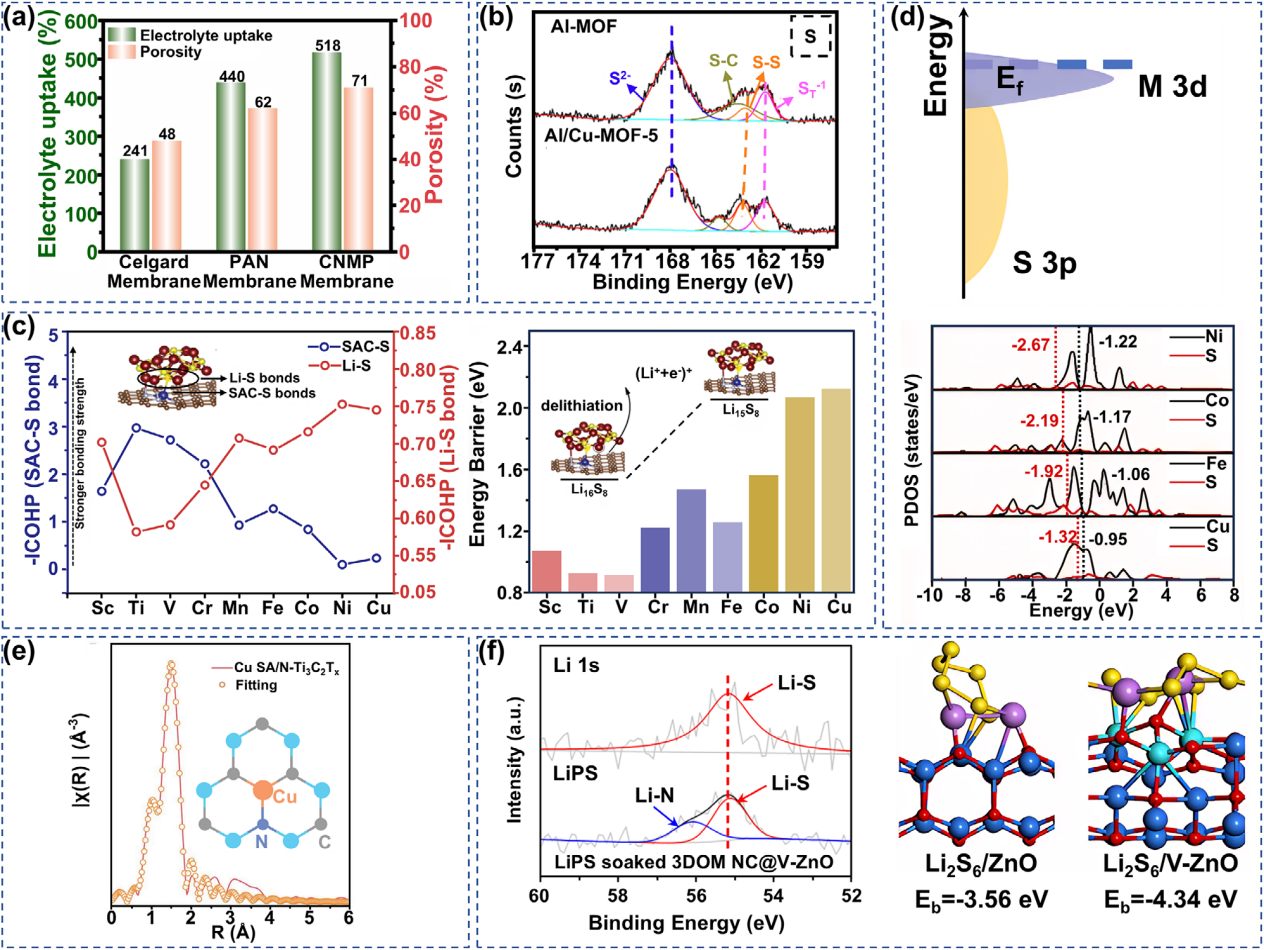
a) Electrolytic absorption and porosity of Celgard, PAN and CNMP. Reproduced with permission.^[^ ^81^ ^]^ Copyright 2024, Elsevier. b) S 2p XPS spectra of Al‐MOF with Al/Cu‐MOF‐5 after LiPSs infiltration. Reproduced with permission.^[^ ^82^ ^]^ Copyright 2021, Wiley‐VCH. c) COHP analysis of the (Li_2_S)_8_‐SAC system and desulfurization energy barriers. Reproduced with permission.^[^ ^83^ ^]^ Copyright 2021, Wiley‐VCH. d) Orbital hybridization mechanism of the M‐N_4_ site and PDOS upon adsorption with Li_2_S_6_. Reproduced with permission.^[^ ^84^ ^]^ Copyright 2023, Elsevier. e) FT‐EXAFS fitting curve of Cu SA/N‐Ti_3_C_2_T_x_. Reproduced with permission.^[^ ^85^ ^]^ Copyright 2023, Wiley‐VCH. f) Li 1s XPS spectra and adsorption configurations of Li_2_S_6_ on ZnO and V‐ZnO, Zn‐blue, V‐cyan, Li‐violet, S‐yellow and O‐red. Reproduced with permission.^[^ ^86^ ^]^ Copyright 2021, Elsevier.

Metal‐organic framework materials (MOFs) are ideal carriers for LSBs due to their highly designable pore structure and precisely controllable chemical environment, and the 3D lattice structure formed by their periodically arranged metal nodes and organic ligands can generate a large number of unsaturated coordination sites; these properties endow MOFs with unique surface chemical activity.^[^ ^79^, ^80^ ^]^ The Diao team developed Cu‐MOFs/Li‐Nafion composite separator. The Lewis acid sites enriched in the homogeneous channels of Cu‐MOFs anchored LiPSs through strong chemical interaction. Meanwhile, the negatively charged ‐SO_3_^‐^ group on Nafion repelled the LiPSs anion and alleviated the migration of LiPSs (Figure 3g).^[^ ^73^ ^]^ Cu MOFs/Li‐Nafion composite separators gave LSBs longer discharge platforms with lower polarization characteristics by virtue of the synergistic effect of pore structure sieving and charge repulsion. Based on the multi‐mechanism synergistic design concept, the researchers further combined multi‐level pore regulation with ion transport channel optimization. For example, Leng et al. constructed Cu‐Ni MOFs/polyacrylonitrile (PAN) composite membranes (CNMP) through a multiscale pore synergistic mechanism, and their electrolyte uptake and permeability were superior to those of the conventional celgard membrane (**Figure** **4**a).^[^ ^81^ ^]^ This is mainly attributed to the fact that the porous nature of PAN greatly enhances the electrolyte storage capacity, while the Cu‐Ni MOFs intercalation further enhanced the permeation pathway through the layered pore system, an optimization that contributed to efficient electrolyte permeation and facilitates Li^+^ transport. In addition to its own use as a MOF material, Cu doping could also play an important role in other MOF materials. For example, Al‐MIL‐53 with metal‐saturated coordination is unable to bind efficiently to LiPSs, and the introduction of Cu can resolve this dilemma. In addition, Geng's group developed Al/Cu‐MOF materials by successfully introducing sulfur‐affinity Cu^2+^ into metal‐organic frameworks through an in situ metal doping strategy while ensuring the crystal morphology of Al‐MOF.^[^ ^82^ ^]^ XPS analysis revealed significant positive shifts in the binding energies of the S─S and S─C bonds due to the Cu^2+^ introduction enhancing the electron cloud density between the metal center and Li_2_S_4_ (Figure 4b). Theoretical calculations further clarified that compared with Al^3+^ fixed by physical adsorption of S_x_^2‐^ due to the steric hindrance effect, the d orbitals of Cu^2+^ formed a stronger d‐p orbital hybridization with the p orbitals of S, resulting in a higher Cu‐S binding energy than Al‐S. This synergistic effect of physical adsorption and chemical bonding builds a multilevel domain‐limiting mechanism to effectively inhibit the shuttling effect of polysulfides, and this work optimizes the design of electrode materials through the synergistic effect of metals, providing a new idea for the development of high‐efficiency S‐host materials.

#### 3.4.3 Copper Single‐Atom Catalysts

The single‐atom catalysts (SACs) can significantly enhance the SRR catalytic activity through the exposed active sites and synergistically optimize the conversion kinetics of LiPSs relying on the extremely high atom utilization, providing a solution to mitigate the capacity decay and shuttle effect of LSBs.^[^ ^87^, ^88^, ^89^ ^]^ However, the lack of a fundamental understanding of the catalyst mechanism and the nature of the materials controlling the catalytic activity will hinder the design and selection of SACs for LSBs. For example, Han et al. proposed that the d‐p orbitals were the key to the difference in catalytic activity of SACs, and transition metals with lower atomic numbers exhibited stronger d‐p orbital hybridization during the adsorption of S species due to their lower degree of antibonding orbital fillings, and this electronic structural property effectively enhanced the stability of the metal‐S bonding.^[^ ^83^ ^]^ By comparing the transition metal Ss, the multiplicative performance of Cu SAC@CF at high S loading was poor, mainly due to the low d‐p hybridization efficiency and the more filled antibonding orbitals of Cu, leading to the weak adsorption and reaction kinetic modulation of LiPSs (Figure 4c). It may still be necessary to enhance its catalytic performance in the future by improving the coordination environment or bimetallic synergistic strategies. Li's group has innovatively designed a series of MOF‐based SACs with M‐N_4_ coordination configurations through the precise tuning of the electronic structure of the d orbitals in the metal center.^[^ ^84^ ^]^ Theoretical calculations showed that the active center of Cu─N_4_ exhibited unique d─p orbital hybridization characteristics. Its d_xz_/d_z_^2^ orbitals formed a strong coupling effect with the p orbitals of LiPSs, resulting in the generation of the minimum d‐p hybridization energy gap in the system. This significantly enhanced the electron interaction between the metal center and Li_2_S_6_. The XPS results confirmed the existence of enhanced chemical adsorption between Cu─N_4_ and LiPSs (Figure 4d). On the basis of the coordinated optimization approach, the Gu team further constructed an asymmetric coordination system through carrier innovation, they successfully developed a d/p‐region metal SACs system anchored to a nitrogen‐doped Ti_3_C_2_T_x_ substrate, and a systematic screening revealed that the Cu‐based catalyst exhibited optimal SRR electrochemical performance.^[^ ^85^ ^]^ X‐ray absorption fine structure spectroscopy (EXAFS) and DFT analyses confirmed that the active center was an asymmetrically coordinated Cu─N_1_C_2_ site, a configuration with higher binding energy and larger electron cloud density of LiPSs, enhancing the adsorption and accelerating the SRR kinetic conversion of LiPSs (Figure 4e). This asymmetric coordination configuration may lead to electron cloud rearrangement and lattice distortion, in turn affecting the adsorption and conversion of LiPSs. In conclusion, Cu SACs are promising for LSBs, but still need to be optimized by improving the coordination environment or introducing bimetals for their application at high S loading.

## 4 Zinc‐Based Catalysts Materials for LSBs

In recent years, Zn‐based compounds have shown unique advantages in the field of cathode materials for LSBs. The fully filled electronic configuration of the Zn element is hybridized with the unsaturated p‐orbitals of S, and its orbital properties induce a multilevel pore structure and abundant coordination of unsaturated sites.^[^ ^90^ ^]^ The unique fully filled electron configuration of Zn leads to the extremely low d‐band center position of Zn. This weakens its adsorption on LiPSs, and the weaker adsorption can effectively avoid the poisoning of the active site caused by strong adsorption and ensure the continuity of the catalytic cycle.^[^ ^91^ ^]^ In terms of the catalytic mechanism, although the Zn‐filled d orbitals are not directly involved in the strong bonding interactions, they can inject electrons into the σ* orbitals of LiPSs during the discharge process, which can significantly weaken the S‐S bond and promote its breakage.^[^ ^92^ ^]^ Compared with classical transition metal catalysts with unfilled d orbitals, Li_2_S is more readily desorbed from the surface of Zn‐based catalysts and the energy barrier for LiPSs conversion is lower. This unique electronic structure enables the Zn‐based materials to effectively balance the moderate anchoring of polysulfides with efficient catalysis, thereby enhancing the overall reaction kinetics.

### 4.1 Zinc Oxide Catalysts

**Figure 5:**
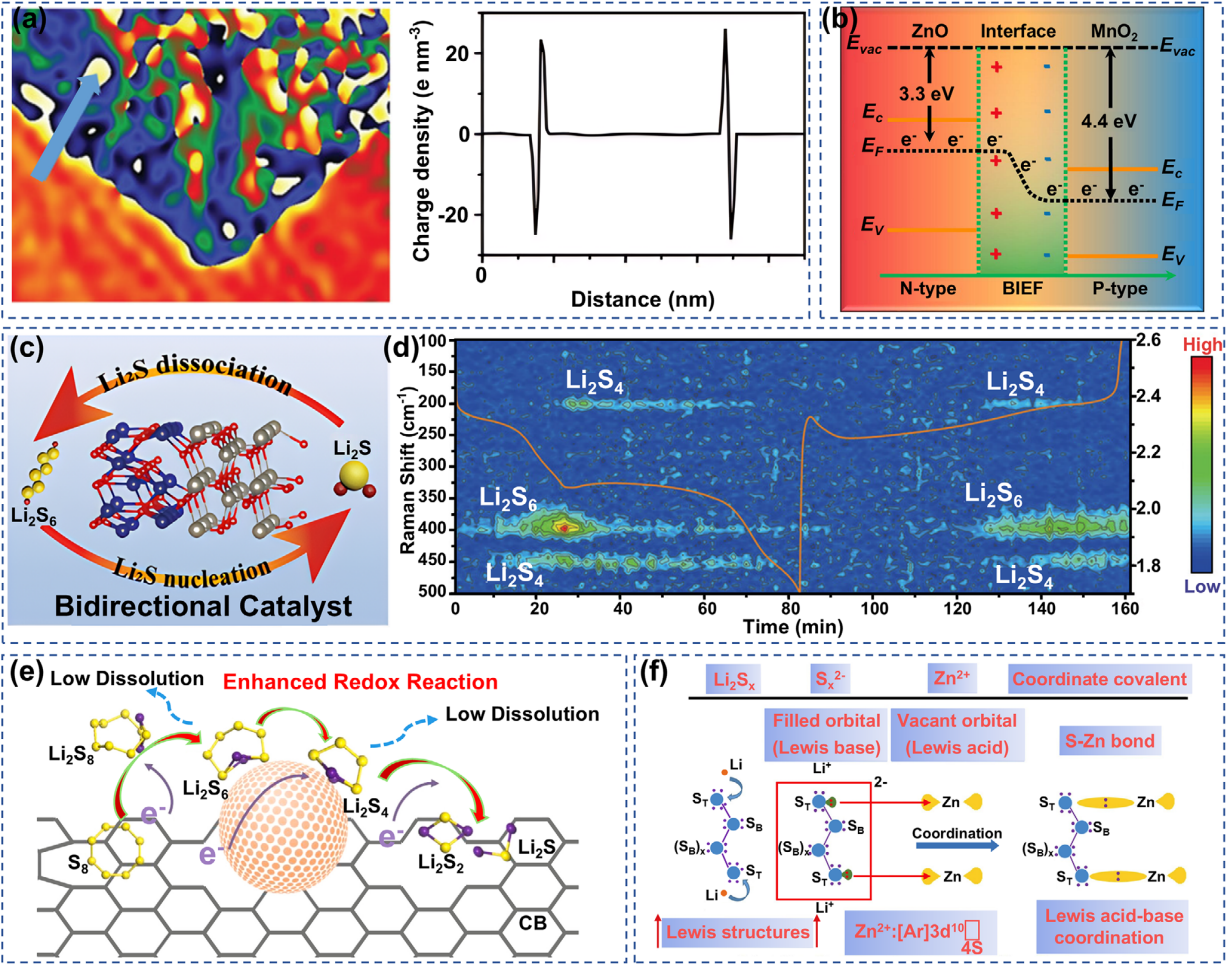
a) Holographic image and localized charge density distribution of ZCCDN. Reproduced with permission.^[^ ^93^ ^]^ Copyright 2020, Wiley‐VCH. b) Schematic of the energy band structure of ZnO/MnO_2_ heterojunction. Reproduced with permission.^[^ ^95^ ^]^ Copyright 2024, Elsevier. c) Schematic representation of the catalyzed by Co_3_O_4_/ZnO catalysts; d) In situ Raman spectra of S@CZO/HNC cathode. Reproduced with permission.^[^ ^97^ ^]^ Copyright 2023, Wiley‐VCH. e) Schematic representation of ZnS‐catalyzed enhancement of the redox reaction of LiPSs. Reproduced with permission.^[^ ^98^ ^]^ Copyright 2018, Elsevier. f) Mechanism of Zn‐S bond formation based on Lewis acid‐base theory. Reproduced with permission.^[^ ^99^ ^]^ Copyright 2020. Elsevier.

ZnO shows significant value in LSB materials due to its unique chemical immobilization, and the polar interfacial properties of the material can be used to inhibit the diffusive migration of LiPSs by forming bonding cooperation with them. On this basis, heterogeneous element doping can synergistically optimize material properties. V‐doped ZnO‐based catalysts with 3D ordered macropores were prepared by Zhao's team.^[^ ^86^ ^]^ XPS spectral analysis revealed that the Li 1s pattern exhibited a characteristic Li‐N peak at 56.1 eV after the adsorption of LiPSs, indicating that the material realized the effective anchoring of LiPSs through the formation of Li‐N chemical bonds. Theoretical calculations further confirmed that the introduction of V enhanced the interaction of the host material with LiPSs (Figure 4f). In addition, the doping of V caused the band gap of ZnO to essentially disappear, and this semiconductor‐metalloid transition established an efficient electron transport channel for S redox reactions. Interfacial charge modulation strategies based on the optimization of electron transport pathways further drive innovation in the design of S‐hosted materials. For example, Xu et al. designed a novel hollow material characterized by ZnO nanoparticles encapsulated within a cobalt‐doped nickel oxide multifaceted framework (ZCCDN).^[^ ^93^ ^]^ They found by electron holographic analysis that there was a local charge redistribution at the interface of cobalt‐doped nickel oxide (CDN) polyhedra with ZnO particles, and positive charges were mainly enriched on the inner surface of CDN and the outer surface of ZnO, forming complementary charge aggregation zones, and this asymmetric charge distribution built the polarized electric field. Specifically, the high negative charge density on the outer surface of CDN provided a driving force for the rapid adsorption and directed migration of Li^+^ (**Figure** **5**a). The large number of interfaces present in the ZCCDN material effectively inhibits the deposition of the insulating layer and mitigates polarization by creating an electric field with high polarization density. It is shown that the design of p‐n junctions can not only form a BIEF through energy band modulation, but also achieve directional charge migration and optimize the adsorption‐catalytic mechanism of LiPSs.^[^ ^94^ ^]^ For example, Yan's research team successfully embedded ZnO nanoparticles and coated a layer of MnO_2_ in lotus‐root‐like porous carbon fibers.^[^ ^95^ ^]^ The system was based on the difference in the figure of merit between n‐type ZnO and MnO_2_, and the spontaneous transfer of electrons was realized through interfacial coupling. Because the work function of ZnO was relatively small, electrons at the interface would be directionally transferred to MnO_2_ with a larger work function until the Fermi energy levels on both sides reached equilibrium. This process caused a redistribution of charges and formed an interfacial electric field. This interfacial electric field could effectively induce the directional migration of LiPSs from the highly adsorbed MnO_2_ to the highly catalytically active ZnO, reducing the diffusion of LiPSs into the electrolyte (Figure 5b). Heterojunctions can be used as a method of interface engineering to synergistically optimize charge transport and redox kinetics through energy band matching and effectively regulate the bidirectional transformation of S species.^[^ ^96^ ^]^ For example, Wang's group constructed hollow N‐doped carbon nanocage (HNC) materials enriched with Co_3_O_4_/ZnO (CZO/HNC) heterojunctions, and electrochemical tests showed that the heterojunctions promoted the bidirectional transformation of S species (Figure 5c).^[^ ^97^ ^]^ Real‐time monitoring by in situ Raman spectroscopy revealed that the intensity of the S_6_^2‐^ characteristic peaks gradually decreases during the discharge process, while reversible recovery was achieved during the charging process, demonstrating the efficient and reversible conversion of LiPSs in S@CZO/HNC (Figure 5d). This efficient reversible conversion helped to achieve high specific capacity and low polarization characteristics. This electric field pushed the directional migration of Li^+^ through the built‐in potential difference at the polar interface. The strength of the spatial distribution of the interfacial electric field can be further enhanced by optimizing the crystal orientation or constructing a heterogeneous structure of ZnO, thereby improving the chemisorption and catalytic conversion efficiency of SRR.

### 4.2 Zinc Sulfide Catalysts

Zn sulfide can inhibit LiPSs shuttling through the synergistic effect of the physically confined domains of the 3D porous structure and Zn‐S bonds. Its surface‐enriched S vacancies and optimized electronic energy band structure can significantly lower the Li_2_S formation energy barrier and accelerate the solid‐liquid‐solid phase transition kinetics of SRR. For example, Xu et al. demonstrated for the first time that ZnS could be used as a catalyst in LSBs by innovatively developing ZnS nanorods with a spherical structure.^[^ ^98^ ^]^ They found that ZnS significantly accelerated the charge transfer process between Li^+^ and LiPSs, and at the same time, the migration activation energy of LiPSs on the ZnS surface decreased. They elucidated from the atomic scale the adsorption‐diffusion‐transformation of LiPSs on the ZnS surface to ensure rapid diffusion of the adsorbed reactive species to the substrate for subsequent reactions (Figure 5e). This study of the SRR catalytic effect, focusing on nano‐structural design, lays the foundation for subsequent in‐depth analysis of the chemical bonding mechanism at the active site. The Li research team embedded ZnS nanoparticles in nitrogen‐doped 3D nanosheets and induced crystal form changes in ZnS through high‐temperature induction. This phase transition promoted the formation of unsaturated Zn^2+^ coordination centers in the material. When ZnS came into contact with LiPSs, Zn^2+^ with the characteristic of electron absence could act as the Lewis acid active site. Through Lewis acid‐base interaction, it formed a Zn‐S coordination covalent bond with S at the end of the long chain of LiPSs that had lone pairs of electrons, achieving anchoring of LiPSs. XPS confirmed the formation of the coordination bond (Figure 5f).^[^ ^99^ ^]^ This chemical bonding mechanism, compared to simple physical adsorption, exhibits a stronger anchoring capacity.

**Figure 6:**
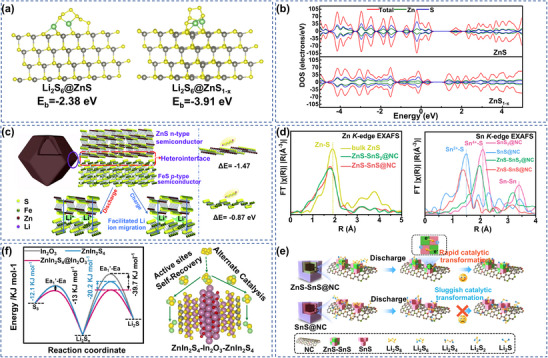
a) Dominant configurations of ZnS and ZnS_1‐x_ for Li_2_S_6_ adsorption. Li‐green; Zn‐gray S‐yellow. Reproduced with permission.^[^ ^101^ ^]^ Copyright 2021, Elsevier. b) DOS analysis of ZnS and ZnS_1‐x_. Reproduced with permission.^[^ ^102^ ^]^ Copyright 2021, Elsevier. c) Synergistic catalytic mechanism of ZnS‐FeS heterostructures. Reproduced with permission.^[^ ^103^ ^]^ Copyright 2020, Royal Society of Chemistry. d) Sn/Zn k‐edge EXAFS spectra of SnS_2_@NC, SnS@NC, ZnS‐SnS_2_@NC, ZnS‐SnS@NC. e) Comparison of S conversion pathways for ZnS‐SnS@NC and SnS@NC. d,e) Reproduced with permission.^[^ ^104^ ^]^ Copyright 2021, American Chemical Society. f) Activation energies for the conversion of S_8_ to Li_2_S_x_ and Li_2_S_x_ to Li_2_S and the catalytic mechanism of ZnIn_2_S_4_‐In_2_O_3_‐ZnIn_2_S_4_. Reproduced with permission.^[^ ^106^ ^]^ Copyright 2023, Elsevier.

The properties of metal sulfides can be tuned and controlled not only by their electron/collector frameworks, but also more significantly by defects in their structures.^[^ ^100^ ^]^ As a typical defect modulation tool, S vacancy engineering can effectively reconfigure the active sites on the surface of the material and realize the anchoring of LiPSs. For example, Gao's team developed hollow‐structured ZnS_1‐x_ composites with S vacancies based on ZIF‐8 templates.^[^ ^101^ ^]^ Comparison of adsorption tests and DFT calculations revealed that the binding energy of Li_2_S_6_ on ZnS_1‐x_ is higher than that of ZnS with intact lattice, indicating that ZnS_1‐x_ has a stronger affinity for LiPSs (**Figure** **6**a). The introduction of such S vacancies not only improves the anchoring ability of the material, but also, by tuning the band gap, promotes electron conduction. Moreover, Wang et al. deposited ZnS_1‐x_ on an independent carbon cloth electrode used for LSBs.^[^ ^102^ ^]^Through density of states analysis, it was confirmed that it exhibited obvious energy gaps in the conduction band and valence band, and had typical semiconductor characteristics. S vacancy‐induced band gap narrowing optimized ion transport and effectively suppressed polarization by enhancing material conductivity (Figure 6b). Defect engineering optimizes the mass transfer channel through the regulation of surface SRR active sites and band structure and can break through the kinetic limitations of LSBs. Based on the synergistic regulation strategy of surface SRR active sites and band structures by defect engineering, Li^+^ mass transfer channels and LiPSs charge transfer pathways were simultaneously optimized by increasing adsorption sites and electron transport networks. Meanwhile, defect engineering not only significantly improves S redox kinetics, but also greatly suppresses the shuttle effect.

Recently, it has been demonstrated that ZnS/metal sulfide heterostructures also exhibit good electrochemical performance in LSBs, and the heterostructures may improve interfacial and surface charge transfer during charging and discharging. For example, Li's group developed an N‐doped carbon‐embedded ZnS‐FeS heterostructure, and the strong BIEF formed at the heterogeneous interface of the material improved the charge transport efficiency.^[^ ^103^ ^]^ Meanwhile, polar ZnS‐FeS had a strong affinity for LiPSs. Through chemical anchoring, LiPSs were confined within N‐doped nanocages, alleviating the migration and diffusion of LiPSs (Figure 6c). The synergistic effect of components in the design of heterostructures is becoming a research hotspot, and the optimization of interfacial charge distribution through energy band engineering can further improve the catalytic performance. Besides, Yao's team prepared ZnS‐SnS@NC catalysts with uniform cubic morphology, and X‐ray absorption fine structure spectroscopy (EXAFS) confirmed the formation of a strongly coupled interface between ZnS and SnS.^[^ ^104^ ^]^ This interfacial coupling enhanced the charge transfer efficiency by tuning the electronic and structural features of ZnS‐SnS (Figure 6d). Experimental and theoretical calculations revealed that the Zn‐S bond formed by ZnS and LiPSs as well as the energy band modulation at the heterogeneous interface acted synergistically to realize the anchoring‐diffusion‐conversion and rapid charge transfer of LiPSs, effectively inhibiting the ineffective deposition of Li_2_S and ensuring the full contact between the active substance and the active substance during the solid‐solid interfacial reaction process (Figure 6e). This heterogeneous structure enables the optimization of charge distribution and efficient conversion of LSBs through the synergistic effect of interfacial energy band modulation and chemical anchoring. The combined effect of the BIEF accelerating charge transfer, polar components anchoring LiPSs, and band engineering regulating the electronic state at the interface has broken the bottleneck of SRR kinetics, providing a reference for the design of highly stable LSBs.

In addition, ZnIn_2_S_4_ (ZIS), as a ternary sulfide derivative of ZnS, promotes reversible oxygen electrode reaction in the cell with the introduction of In element compared to monometallic sulfides, and ZnIn_2_S_4_ exhibits strong chemisorption and strong catalytic activity for LiPSs. For example, Zhang et al. creatively introduced ZIS@C@S electrodes derived from zeolitic imidazolate framework‐8 (ZIF‐8), and thanks to polar ZnIn_2_S_4_ nanosheets, the migration of LiPSs was limited and the reaction kinetics were improved.^[^ ^105^ ^]^ It is different from ordinary heterostructures. Besides, Jiao et al. prepared ZnIn_2_S_4_‐In_2_O_3_‐ZnIn_2_S_4_ sandwich structures with self‐repairing ability; the ZnIn_2_S_4_ network in the outer layer preferentially adsorbs S_8_, and the In_2_O_3_ in the middle serves as an adsorption medium to promote the conversion of S_8_ to long‐chain Li_2_S_6_/Li_2_S_4_, while the ZnIn_2_S_4_ in the inner layer further catalyzes the transformation of long‐chain Li_2_S_6_/Li_2_S_4_ to short‐chain Li_2_S_2_/Li_2_S. Simultaneously, the self‐recovery of the catalyst can be realized during the charging and discharging process. This design not only significantly improved the cycling stability of LSBs, but also provided innovative ideas for the development of multifunctional catalysts capable of precisely regulating the redox reactions of LiPSs (Figure 6f).^[^ ^106^ ^]^ In conclusion, ZnS has hysteresis in charge transfer kinetics due to its inherent semiconductor properties, and it is still necessary to optimize the electronic structure, reduce the Li^+^ diffusion barrier, and enhance the SRR kinetics and cycling stability through defect engineering or introduction of heterostructures in the process of practical application.

### 4.3 Zinc Selenide Catalysts

**Figure 7:**
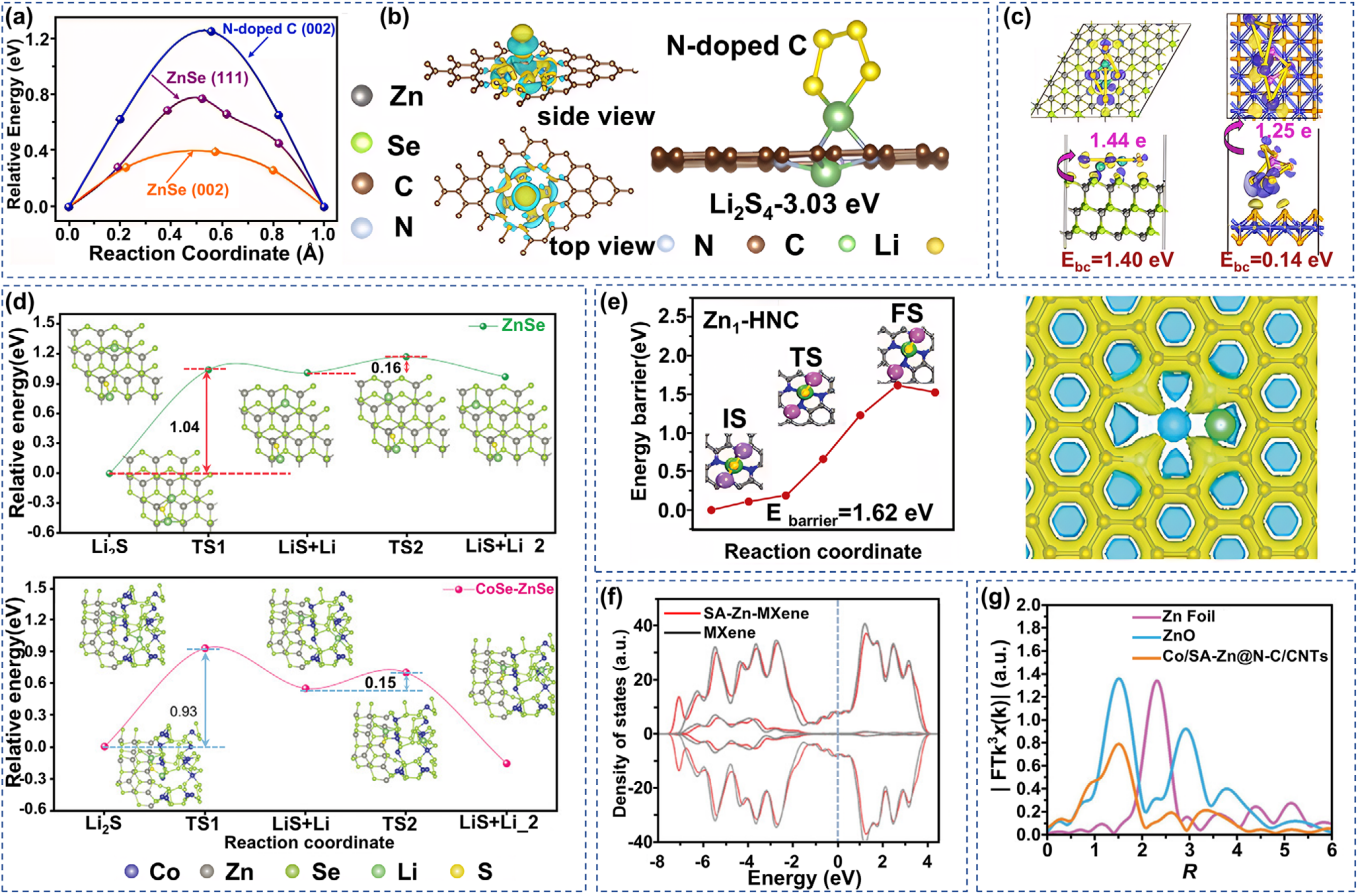
a) Migration energy distribution of Li_2_S_4_ at different crystal planes of ZnSe. b) Charge density difference of ZnSe/NHC and adsorption configuration of Li_2_S_4_ on N‐doped C surface. (a,b) Reproduced with permission.^[^ ^110^ ^]^ Copyright 2020, American Chemical Society. c) Top and side views of the charge density distribution of Li_2_S_4_ on the ZnSe (111) surface and Li_2_S_6_ on the CoSe (110) surface. The dark blue part is the charge accumulation area and the light yellow color is the charge reduction area. Reproduced with permission.^[^ ^112^ ^]^ Copyright 2023, Elsevier. d) Schematic of Li^+^ diffusion at the heterogeneous interface of ZnSe and CoSe‐ZnSe. Reproduced with permission.^[^ ^113^ ^]^ Copyright 2021, Wiley‐VCH. e) Decomposition energy barriers of Li_2_S on Zn_1_‐HNC and HNC and deformation charge densities of Zn_1_‐HNC at the adsorption sites of Li atoms. Reproduced with permission.^[^ ^117^ ^]^ Copyright 2020, Wiley‐VCH. f) DOS of SA‐Zn‐MXene. Reproduced with permission.^[^ ^118^ ^]^ Copyright 2020, Wiley‐VCH; g) Zn k‐edge FT‐EXAFs for Co/SA‐Zn@N‐C/CNFs, ZnO and Zn foils. Reproduced with permission.^[^ ^119^ ^]^ Copyright 2022, Wiley‐VCH.

Compared with metal oxides and sulfides, metal selenides combine higher bulk capacity with heterogeneous interfacial SRR catalytic activity. ZnSe has sulfide‐anchored properties and its hierarchical pore structure facilitates electrolyte wetting, making it an ideal choice as a high‐performance host material for LSBs.^[^ ^107^, ^108^, ^109^ ^]^ For example, Yang's research team innovatively constructed a ZnSe/N‐doped hollow carbon (ZnSe/NHC) composite catalyst.^[^ ^110^ ^]^ The (002) crystal surface of ZnSe exhibited a lower Li^+^ diffusion energy barrier, significantly improving the Li^+^ migration efficiency (**Figure** **7**a). In addition, DFT calculations revealed that electrons were transferred from ZnSe to NHC and clustered around N atoms, and this electronic reconfiguration not only enhanced the overall electrical conductivity, but also realized the effective immobilization of LiPSs through the chemical anchoring effect of Li‐N bonds (Figure 7b). This triple synergistic mechanism of physically confined domains‐chemical adsorption‐catalytic conversion significantly enhances S utilization, suppresses the shuttle effect and improves the SRR kinetics, providing an innovative solution to realize LSBs with high energy density and long cycle life.

Similar to ZnS, heterostructures consisting of ZnSe and other metal selenides have also been reported, and CoSe_2_ has been used in LSBs due to its metal‐like conductivity and excellent catalytic activity. Liu et al. embedded ZnSe@CoSe_2_ heterostructures into nitrogen‐doped carbon nano‐arrays grown on carbon cloth (CC), and the ZnSe@CoSe_2_ hetero‐interface ensured the smooth adsorption‐diffusion transformation process of LiPSs and induced the 3D radial growth of Li_2_S_1/2_ during the cycling process.^[^ ^111^ ^]^ Similar to CoSe_2_, the heterostructure formed by CoSe and ZnSe can also effectively promote the S redox reaction; ZnSe‐CoSe heterojunction hollow nitrogen‐doped carbon nanocages developed by Feng's team realize efficient capture of LiPSs through dual anchoring mechanism.^[^ ^112^ ^]^ Besides, DFT confirmed that chemical bonding between Li in Li_2_S_4_ and Se in ZnSe as well as electrostatic interactions between S and Zn synergistically anchor the LiPSs. Meanwhile, the charge transferred between Co‐S enabled the construction of a localized charge‐rich region on the surface of LiPSs, realizing the local concentration of charge (Figure 7c). This chemical bonding‐charge enrichment synergistic mechanism enhanced the adsorption‐catalytic effect on LiPSs. Structural innovation of heterojunctions not only focuses on the optimization of adsorption mechanism, but also reconfigures charge transport pathways through energy band engineering, opening up new dimensions for catalytic activity enhancement. For example, Ye et al. designed CoSe‐ZnSe heterojunctions to achieve dual optimization of energy band structure and solid‐liquid reaction kinetics.^[^ ^113^ ^]^ DFT calculations showed that ZnSe exhibits semiconducting properties, while the CoSe‐ZnSe heterostructure significantly enhanced the electronic conduction by forming metallic properties through energy band modulation, and this energy band engineering effectively reduced the charge transfer impedance. More importantly, the heterogeneous interface acted as a catalytic active center to reduce the energy barrier for Li_2_S decomposition from 1.04 to 0.93 eV, accelerating the conversion efficiency of Li_2_S solid phase to liquid LiPSs during the charging process (Figure 7d). Based on the research foundation of heterogeneous structure optimization, the introduction of defect engineering provides a new direction for active site design. Meanwhile, Zhou's team introduced Se‐rich vacancies based on the guaranteed heterogeneous and hollow structure, and such vacancies provided binding sites to capture LiPSs and catalyze both long and short chain LiPSs consecutively and efficiently.^[^ ^114^ ^]^ Experiments and theoretical calculations confirmed that ZnSe and SnSe_2_ can realize the baton‐like transformation of LiPSs.

### 4.4 Zinc Single‐Atom Catalysts

In addition to the above compounds, SACs show unique advantages in LSBs by virtue of their maximized atom utilization, fusion of homogeneous and non‐homogeneous catalytic properties, excellent catalytic activity, and LiPSs anchoring ability.^[^ ^115^, ^116^ ^]^ For example, Shi et al. developed SA Zn‐modified hollow carbon spheres, and theoretical calculations showed that the Zn‐N_4_ coordination structure significantly lowered the Li_2_S decomposition energy barrier and effectively accelerated the electrochemical reaction of the delithiation process. Meanwhile, the Li atoms exhibited a significant overlap of the electron cloud around the single‐atom Zn sites, and this electron cloud distribution was more favorable for the uniform deposition of Li, and these results were in agreement with the experimental results (Figure 7e).^[^ ^117^ ^]^ This unique atomic‐level interface modulation strategy effectively reduced the shuttling effect. Altered electron cloud distribution may further trigger broader energy band restructuring, prompting researchers to explore the effects of SA modifications on overall electronic structure. For example, Zhang's team constructed a SA Zn‐modified MXene through an atomic‐level dispersion strategy. The introduction of Zn atoms significantly changed the electronic structure of MXene, and the density of states distribution showed an obvious broadening trend near the Fermi energy level, contributing to the increase of valence band energy level (Figure 7f).^[^ ^118^ ^]^ The enhancement of the energy level structure reduces the electronic excitation barrier, making the valence band electrons more susceptible to excitation into higher energy states, thus facilitating charge transfer during SRR catalysis. This SA‐induced localized electronic excitation effect may provide a new design idea for the construction of adaptive catalytic interfaces by dynamically regulating the adsorption‐desorption equilibrium adsorption strength and catalytic dissociation rate of SRR.

**Figure 8:**
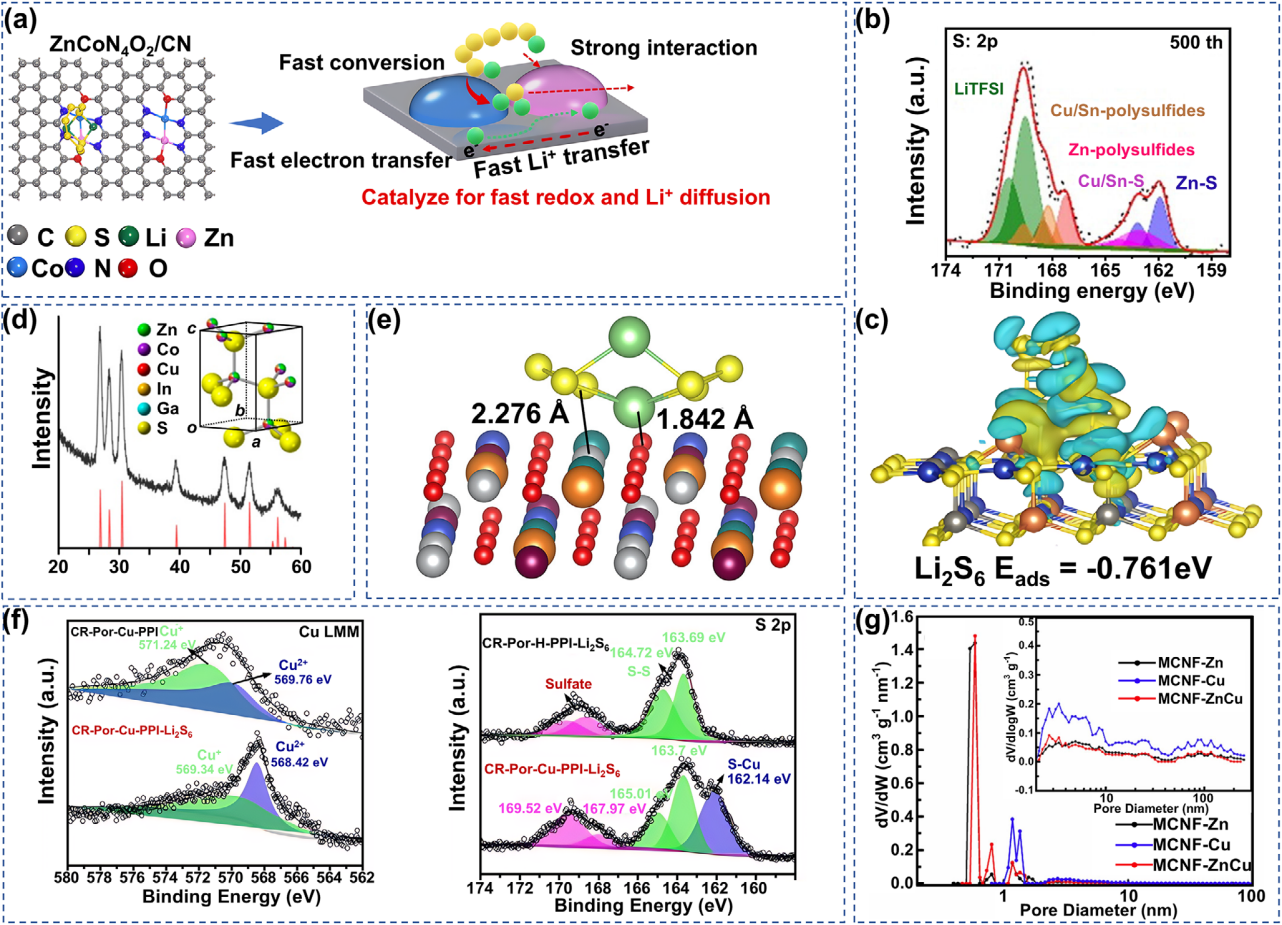
a) Working principle of binuclear monatomic ZnCoN_4_O_2_. Reproduced with permission.^[^ ^120^ ^]^ Copyright 2022, Elsevier. b) In situ XPS fine spectra of CZSS electrode after 500 cycles. c) Difference in charge density of Li_2_S_6_ adsorbed configuration on the CZSS (002) surface. Cyan and yellow regions represent electron loss and gain, respectively. b,c) Reproduced with permission.^[^ ^129^ ^]^ Copyright 2020, Elsevier. d) XRD comparison of Zn_0.30_Co_0.31_Cu_0.19_In_0.13_Ga_0.06_S nanoparticles (black) with fibrillar zincite (red). Fibrillar zincite single cell (a = 3.83 Å, c = 6.29 Å) is shown in the accompanying figure. Reproduced with permission.^[^ ^133^ ^]^ Copyright 2023, American Chemical Society. e) Geometric configurations of Li_2_S_6_ bound to HEMO‐1 (O‐red; Ni‐white; Mg‐orange; Zn‐green; Cu‐blue; Co‐violet; Li‐bright yellow; S‐light green). Reproduced with permission.^[^ ^134^ ^]^ Copyright 2019, Elsevier. f) XPS spectra of N 1s/S 2p of CR‐Por‐Cu‐PPI and CR‐Por‐Cu‐PPI‐Li_2_S_6_. Reproduced with permission.^[^ ^135^ ^]^ Copyright 2021, Elsevier. g) pore size distribution of MCNF‐Zn, MCNF‐Cu, MCNF‐ZnCu. Reproduced with permission.^[^ ^136^ ^]^ Copyright 2024, Elsevier.

Different from the SA modification system, the nano‐atomic multi‐scale active centers can realize the gradient modulation of electronic structure and multiple complementary active sites. For example, Wang's group constructed a Co/SA‐Zn@N‐C composite catalyst system by a bimetallic template strategy.^[^ ^119^ ^]^ Synchrotron radiation showed that the complete absence of the characteristic peaks of the Zn‐Zn metallic bond directly confirmed that Zn stably existed in the nitrogen‐doped carbon substrate in the form of atomic‐level dispersion, and this atomic‐level dispersion property made the Zn‐N_4_ formed by Zn and N have more exposed active sites, and at the same time, Co nanoparticles produced electronic synergy with the atomically dispersed Zn‐N_4_ sites, enhancing the Li_2_S_2_ to Li_2_S transformation (Figure 7g). Unlike the electronic synergistic effect of metal‐monoatomic in heterogeneous structures, the homogeneous diatomic system further strengthens the interfacial coupling. For example, Song's research team developed a carbon nanosheet (CN)‐loaded binuclear SACs ZnCoN_4_O_2_/CN.^[^ ^120^ ^]^ Studies have demonstrated that Zn sites anchored Li^+^ and accelerated its migration through strong lipophilicity, while Co sites promoted the adsorption transformation of LiPSs by virtue of their nucleophilicity, and that the Zn‐Co pair was stabilized as a diatomic form in the carbon matrix. Electron density difference analysis showed that a strong covalent bond was formed at the interface, allowing ZnCoN_4_O_2_/CN to retain its atomic coordination structural integrity even after many lithiation/delithiation cycles (**Figure** **8**a). This dual active center mechanism broke through the monocenter limitation of traditional monoatomic catalysts and constructed an ion‐anchoring‐electron‐transporting bifunctional interfacial structure through charge synergism and spatial coupling effects between heteronuclear metals, significantly improving catalytic kinetic performance. In conclusion, the monometallic Zn catalysts achieve a high density of active sites through atomic‐level dispersion, and their optimized electronic structure promotes Li‐S bonding and charge transfer.

The differences between copper‐based and zinc‐based materials in catalyzing the conversion mechanism of LiPSs stem from their unique electronic structures. Copper has the configuration [Ar]3d^10^4s^1^. Its d‐band is full, but its 4s^1^ electron delocalizes easily. This gives copper moderate, and moderate adsorption strength. In contrast, zinc has the configuration [Ar]3d^10^4s^2^. Its d‐band is full and 4s orbitals are fully occupied. This results in lower electronegativity and strong Lewis acidity, making zinc primarily an electron acceptor. Furthermore, the position of the d‐band center further controls LiPSs adsorption. Copper's d‐band center is close to the Fermi level, allowing it to form medium‐strength adsorption with LiPSs anti‐bonding orbitals. This balance effectively manages LiPSs activation and desorption. Zn d‐band center is far from the Fermi level, which significantly enhances its d‐orbital localization. Consequently, in catalysis, Cu‐based materials adsorb LiPSs moderately, effectively anchoring the molecules without blocking later reactions. Zn‐based materials, however, use their strong Lewis acidity. They preferentially stabilize the terminal sulfur atoms in LiPSs.

## 5 Copper‐Zinc‐Based Catalysts Materials for LSBs

The Cu‐Zn bimetallic catalysts system was constructed as an efficient LiPSs synergistic catalytic conversion network through the complementary effect of electronic coupling and spatial site resistance between Cu/Zn atoms. Cu sites optimize the charge transport path through electronic interactions and significantly reduce the interfacial impedance, while Zn sites preferentially anchor higher‐order LiPSs and inhibit their dissolution and diffusion by virtue of their higher electronegativity. In order to analyze the material realization path of this co‐design, some typical Cu‐Zn matrix composites will be listed in this section.

### 5.1 Copper‐Zinc Sulfide Catalysts

Compared with monometallic sulfides, bimetallic sulfides can optimize charge transport pathways through synergistic interactions, significantly enhancing the kinetics and cyclic stability of redox reactions. Heterostructure design is one of the main approaches to fundamentally manipulate the electronic structure of polar materials.^[^ ^121^, ^122^ ^]^ According to the d‐band center theory, this tensile or compressive strain will trigger the upward or downward shift of the center of the metal band, thereby directly regulating the adsorption behavior of the catalyst for intermediate products.^[^ ^123^, ^124^ ^]^ Specifically, the upward shift of the d‐band center enhances d‐p orbital hybridization between metal atoms and S atoms in LiPSs. The hybridization raises antibonding orbital energy levels, strengthening LiPSs adsorption.^[^ ^125^ ^]^ Meanwhile, the synergistic interaction between different components at the heterogeneous interface significantly accelerates the diffusion and transfer of ions and electrons, while the interface‐induced high‐density lattice defects and more extensive electronic structure reconstruction further optimize the interfacial charge transfer process. These synergistic effects of microscopic electronic structure changes and interfacial effects brought about by heterostructure design ultimately lead to significant enhancement of electrochemical kinetics and potentially unique and novel catalytic properties. For example, Zhai et al. fabricated a p‐Cp/CuS/ZnS composite catalyst via continuous impregnation, using Canadian pine wood‐derived porous carbon (p‐Cp) as a carrier.^[^ ^126^ ^]^ The core of this strategy lies in the construction and regulation of the CuS/ZnS bimetallic interface, with lattice strain inevitably introduced from the inherent difference in lattice constraints between CuS and ZnS.^[^ ^127^ ^]^ Specifically, lattice strain can optimize the position of the d‐band center of CuS to enhance its electron transfer ability as a redox mediator, as well as modulate the density of states near the Fermi energy level of ZnS to strengthen its chemisorption affinity for LiPSs and reduce the reaction energy barrier. The p‐type CuS acted as a redox mediator to accelerate electron transfer and promoted the liquid‐solid conversion of LiPSs, and n‐type ZnS adsorbed LiPSs through strong chemisorption and lowered the reaction energy barrier. The sustainability of biomass carbon and the dynamic adsorption‐catalytic function of the composite SRR catalyst provides a low‐cost solution for high‐capacity LSBs. In addition to heterogeneous structural synergies, the spatial distribution of SRR catalytic activity and active sites is further optimized through quantum domain‐limiting effects. For example, Artchuea's group constructed CuZnS quantum dot‐modified (NiCo)‐S/graphene‐CNT composite cathode by hydrothermal method.^[^ ^128^ ^]^ The design took full advantage of the unique quantum confinement property of quantum dots, and the high specific surface area brought by the nanoscale significantly increased the exposure of active sites, effectively increasing the chemisorption of LiPSs. On the basis of CuZnS binary metal sulfide, the addition of Sn enhanced the decomposition of Li_2_S. For example, Zha's research team designed and synthesized 2D Cu‐Zn‐Sn polymetallic sulfide nanosheets.^[^ ^129^ ^]^ In situ XPS tests showed that the Cu 2p binding energy of the electrode remained stable after cycling, confirming the anchoring of long‐chain LiPSs at the Cu site via strong Cu‐S covalent bonds, while the Sn 3d and Zn 2p characteristic peaks were not shifted, indicating a stable chemical state on the surface of the material (Figure 8b). Combined DFT calculations revealed that the Cu‐S bond exhibited strong adsorption properties due to high electron cloud overlap, while the Sn/Zn sites synergistically promoted the catalytic conversion of LiPSs through moderate electron localization (Figure 8c). This synergistic catalytic design of multi‐metal sites not only embodied the hierarchical regulation strategy of Cu sites to achieve strong immobilization of LiPSs and Sn/Zn sites to accelerate the subsequent redox reaction, but also constructed a synergistic catalytic interface with multiple active sites, providing a new paradigm of interfacial engineering for solving the shuttling effect of LSBs. High‐entropy sulfides have advantages in the development of advanced electrocatalytic materials. The interaction among their multiple components can produce a synergistic effect, providing a variety of chemical and electronic adsorption sites for catalyzing complex reactions.^[^ ^130^, ^131^, ^132^ ^]^ Theibault et al. synthesized high entropy sulfide Zn_0.30_Co_0.31_Cu_0.19_In_0.13_Ga_0.06_S nanoparticles by multiple exchanges of Cu_1.8_S nanoparticles.^[^ ^133^ ^]^ The X‐ray diffraction (XRD) results show that the lattice parameters are between CoS and CuInS_2_ and are consistent with the weighted average values, indicating that all metal elements are distributed in the sulfide lattice, forming a single‐phase structure (Figure 8d). This single‐phase structure indicated that there was no obvious lattice distortion inside the material, favorable for maintaining the integrity of nanoparticles in electrochemical reactions, and the uniform single‐phase structure could provide abundant active sites to promote the redox reaction of Li_2_S_6_. In brief, Cu‐Zn sulfides significantly enhanced SRR kinetics through bimetallic modulation, but further improvement of the catalytic activity by replacement or addition of other metals may still be required.

### 5.2 Copper‐Zinc Oxides Catalysts

Cu‐Zn synergism in Cu‐Zn oxides effectively inhibits the shuttle effect of LiPSs in LSBs by the triple mechanism of chemical anchoring, electrocatalysis and structural stabilization. Numerous studies have shown that heterogeneous structures formed by nanomaterials with different work functions can generateBIEF that enhance electron/ion transport and surface reaction kinetics.^[^ ^137^, ^138^, ^139^ ^]^ Meanwhile, the heterogeneous structure significantly enhances the electron transfer efficiency, redox kinetics, and adsorption capacity of LiPSs through the synergistic effect between the components. The BIEF can drive the directional migration of LiPSs from the adsorbent side to the catalyst side at the interface of heterogeneous structures.^[^ ^140^ ^]^ This rational structure configuration ultimately realizes a sequential “capture, directed migration, and conversion” reaction mechanism for LiPSs, first enriching them in the adsorbent component, then migrating them to the heterogeneous interface driven by BIEF, and finally converting them to the final product sustainedly on the catalyst surface.^[^ ^141^, ^142^, ^143^ ^]^ For example, ZnO and CuO form a p‐n junction heterogeneous interface with a strong BIEF.^[^ ^144^ ^]^ As a major factor affecting energy storage performance, this BIEF efficiently drives the directional migration of electrons/ions to significantly accelerate LiPSs conversion kinetics.^[^ ^145^ ^]^ Additionally, the opposing charge characteristics on both sides of the interface enable a regionalized division of labor based on charge distribution differences, directly strengthening the synergistic anchoring and catalytic mechanism. Yang et al. constructed the ZnO‐CuO heterointerface.^[^ ^146^ ^]^ The BIEF of the p‐n junction formed by this interface could accelerate the LiPSs transformation. Theoretical calculations revealed that CuO exhibited stronger chemisorption for Li_2_S_6_, while ZnO had higher binding energy for Li^+^, verifying the partitioning mechanism of the heterojunction. Based on the partitioning mechanism of heterojunctions, high‐entropy materials (HEM) achieve a more comprehensive synergistic anchoring through multimetallic compositions, extending the structural advantages from binary to multimetallic systems. Zheng's group innovatively used five transition metals (Ni, Mg, Cu, Zn, Co) to construct high‐entropy metal oxides (HEMO), the material was thermodynamically and chemically stable, contributing to the stable operation of the battery.^[^ ^134^ ^]^ Ni‐O in its lattice could form Li‐O and S‐Ni double chemical bonds with LiPSs through a synergistic mechanism, while the anchoring ability and conversion efficiency of LiPSs were significantly improved by the catalytic synergistic effect of multi‐metal components (Figure 8e). In short, binary and poly copper‐zinc oxides show significant synergistic effects in LSB systems, effectively improving the performance of the batteries with good research value.

### 5.3 Other Copper‐Zinc‐Based Catalysts

Based on the research of copper‐zinc sulfide and oxide systems, the Shi team further broke through the limitations of the traditional inorganic framework and innovatively combined the porphyrin‐based organic porous polymer (CR‐Por‐PPIs) with Cu/Zn/Co polymetallic salts.^[^ ^135^ ^]^ The hierarchical pore structure effectively alleviated the volume expansion. Meanwhile, the chelated metal salt and the polarity N of the porphyrin ring formed strong chemical bonds of N‐Li and S‐Cu with LiPSs, suppressing the shuttle effect. A unique π‐conjugated skeleton with Cu, Zn, and Co chelated metal salts synergistically constructs a 3D conductive network to accelerate the redox kinetic transformation of LiPSs (Figure 8f). By distinguishing between polymetallic salts and organic polymer advantages, Zhao's group introduced zinc and copper salts to construct hierarchical porous structures. Based on the dual‐salt synergistic strategy, they constructed hierarchical porous carbon nanotube fibers using electrospinning technology. Zinc salts induced the formation of microporous networks to effectively anchor S_2_‐S_4_ molecules, while the introduction of copper salts constructed mesoporous channels, significantly enhancing the wettability of the electrolyte and the ion mobility. The synergy of Zn and Cu enabled the material to have a high specific surface area of 545.9 m^2^ g^−1^ (Figure 8g).^[^ ^147^ ^]^ This pore architecture enabled both small molecule S encapsulation and fast reaction kinetics. Unlike the above‐mentioned two‐salt synergistic enhancement of S immobilization for ion transport, the Ngo team utilized the multistage adsorption mechanism of MOF to inhibit the migration of LiPSs. They developed a Cu‐ZIF‐8 functional layer on commercial glass fiber (GF), and the doping of Cu generated additional active sites.^[^ ^136^ ^]^ The improved separator increased the initial capacity of the battery by nearly two times at low multiplication rates compared to the conventional GF. The copper‐zinc bimetallic material significantly improves the anchoring ability of polysulfides in LSBs through an adsorption‐catalytic synergistic mechanism and shows unique advantages in optimizing the interfacial reaction kinetics. However, during long‐term cycling, the dynamic dissolution behavior of the metal active sites triggers lattice structure remodeling, and the superimposed electrode/electrolyte interface side reactions are intensified, leading to irreversible capacity decay. In the future, it may be necessary to improve the carrier transport efficiency by constructing a 3D hierarchical conductive network, optimize the charge distribution by using heterogeneous interface engineering, or develop functional electrolyte systems to construct stable solid‐liquid interfaces, so as to achieve systematic enhancement of material properties.

## 6 Conclusions and Outlook

This paper reviews the latest research progress of 3d^10^‐based materials as LSBs cathode materials. 3d^10^‐based materials include Cu‐based materials, Zn‐based materials, and Cu‐Zn‐based materials, and their applications in LSBs cathode show unique advantages: 1) Cu‐based materials have excellent electron transfer properties to build efficient 3D conductive networks, improving the electron transfer efficiency of the cathode, and their polar surfaces can anchor LiPSs through strong chemisorption; 2) Zn‐based materials can immobilize LiPSs by a dual mechanism of chemisorption and physically confined domains, and they are highly electrochemically active to catalyze SRR; 3) Cu‐Zn based materials form a 3D conductive/ionic network due to the combination of Cu high electrical conductivity and Zn ionic conductivity, enhancing the utilization of S; meanwhile, copper in the Cu‐Zn interface accelerates the electron transfer and Zn promotes the decomposition of LiPSs, significantly enhancing the reaction kinetics. In addition, the Cu‐Zn matrix composites can jointly inhibit LiPSs shuttling through chemisorption in the physically confined domain.

Despite the many advantages of Cu‐Zn based materials, there are a number of areas to be aware of: 1) Cu‐based materials face two major problems. Firstly, Cu‐based materials are prone to interfacial side reactions to generate a CuS_2_ insulating layer, dramatically increasing interfacial impedance. To address the problem of side reactions, a protective polymer layer with a 3D network can be introduced to realize the physical hindrance of the Cu‐S interface. In addition, a carbon‐based cladding layer can be constructed to dual‐regulate the reduction of interfacial impedance and LiPSs migration by virtue of its high electrical conductivity and physically domain‐limiting effect. Secondly, the catalytic efficiency of Cu‐based materials for SRR/SOR is low compared to noble metals. For the problem of poor catalytic performance, the catalytic activity can be improved by introducing transition metals to construct metal synergistic systems, or by defect engineering or constructing heterogeneous structures. 2) Zn‐based materials exhibit insufficient electrical conductivity, triggering sluggish SRR/SOR, while the restricted electron transport path will affect their performance at high magnification. To address the above bottlenecks, the following strategies can be optimized. On the one hand, controlled defect engineering can activate the local electronic structure on the surface of the material to establish an efficient charge transfer channel. On the other hand, the design of heterogeneous interfacial structures can accelerate the directional migration of electrons by virtue of the BIEF. In addition, a 3D conductive network can be constructed by combining the Zn‐based materials with the highly conductive carbon‐based materials to realize continuous electron conduction. 3) The interfacial compatibility control and large‐scale preparation technology of CuZn‐based composite system are not yet mature, and there are fewer related studies, and the improper design of CuZn ratios or structures will lead to electron/ion transport limitation, in this regard, attention should be paid to the ratio of the metal elements and structural design during the preparation process, in the structural design level, the construction of 3D porous frameworks can be optimized through the metal parts of the electronic coupling effect of charge distribution; in the components of the regulation of rare earth, elements can be introduced to improve the charge transfer efficiency through 4f/3d orbital synergy. Regulation, rare earth elements can be introduced to improve the charge transport efficiency through 4f/3d orbital synergy. 4) Porous nanostructures may enable high S‐loading cathodes, but too many porous structures will consume too much electrolyte, reducing the energy density and too many porous structures may affect the structural stability of the cathode. Therefore, it is crucial to explore the proper balance between porosity and S loading. 5) Relevant reaction mechanisms and electrochemical theories are not yet sufficient, and the interaction and electrochemical mechanisms between host materials and SRR are vague. In the future, research could focus on the reaction mechanisms of 3d^10^ metallic materials. New host materials and their composites with 3d^10^ electronic configurations can be systematically explored and investigated to understand the intrinsic influence of their unique d^10^ electronic structures on LiPSs adsorption behavior, electrochemical conversion pathways, and catalytic efficiency. Concurrently, the combination of high‐precision computational simulations and in situ technologies enables deep analysis of the real‐time evolution of S species in complex interfacial environments, key reaction intermediates, and catalytic activity center conformational relationships. This provides a solid theoretical foundation for the precise design and improvement of SRR/SOR.

Although the current research on the use of 3d^10^‐based materials as cathodes for LSBs continues to advance, there is still a large gap between the current research and industrialized applications. In order to improve its performance, more and more researchers are exploring various strategies, including the construction of 3D conductive networks, defect engineering, and heterogeneous interfaces, and we believe that through further research, 3d^10^‐based materials will make a significant breakthrough in LSBs capacity and stability, and become a strong candidate for LSBs cathode materials.

## Conflict of Interest

The authors declare no conflict of interest.
